# Prevalence of Childhood Short Stature, Underweight, Overweight, and Obesity Among Primary School Children

**DOI:** 10.7759/cureus.19651

**Published:** 2021-11-16

**Authors:** Houda Bouali, Khadija Boujtat, Amine Ezzerrouqui, Youssef Lazreg, Siham Rouf, Naima Abda, Hanane Latrech

**Affiliations:** 1 Department of Endocrinology and Diabetology, Mohammed VI University Hospital, Medical School, Mohamed the First University, Oujda, MAR; 2 Laboratory of Epidemiology, Clinical Research and Public Health, Mohamed the First University, Oujda, MAR

**Keywords:** short stature, underweight, overweight, obesity

## Abstract

Background

This study, the first of its kind in Morocco, was conducted to compare the prevalence of growth disorders among children enrolled in public, private, urban, and rural schools in Oujda-Angad Province.

Methods

A cross-sectional observational study was conducted among primary schools in Oujda-Angad Province from November 2017 to April 2018, with a stratified random cluster sampling of public, private, urban, and rural primary schools.

Results

A representative sample of 1582 students aged from five to 15 years old was selected. The sample included 779 females (49.2%) and 803 males (50.8%), with an average age of 9.3 ± 1.96 years. The average weight of the students was 29.3 ± 9.6 kg (range: 12-130 kg), the average height was 133.7 ± 12.16 cm (range: 104.5-175.5 cm), and the mean body mass index (BMI) was 16.05 ± 3.31 kg/m^2^ (range: 8.33-76.9 kg/m^2^). Overall, short stature (SS) and underweight were significantly more prevalent in the public and rural schools, while obesity was highest in the urban private schools that ranked as the schools with the highest socioeconomic status (SES) students (p < 0.01). Female students were more likely to be overweight and obese, while male students were more likely to be underweight.

Conclusion

Our study provides an estimate of the prevalence of excess weight, underweight, and short stature in a primary school population. Our results reflect the importance of the problem, the need to monitor the nutritional status at both the individual and the community level, and the need to put in place preventive, diagnostic, and early management strategies before the problem worsens.

## Introduction

Natural growth is an important indicator of health in childhood. Growth retardation can result from systemic and endocrine diseases or poor diets. In 2019, according to the United Nations Children’s Fund (UNICEF), the World Health Organization (WHO), and the International Bank for Reconstruction and Development/World Bank, 144 million children under five years old were suffering from short stature (SS) worldwide, among which 5.1 million were in Northern Africa [1]. Conversely, the prevalence of childhood overweight and obesity is increasing in all regions of the world. According to the UNICEF/WHO/World Bank Group Joint Child Malnutrition Estimates, there are nearly 38.3 million overweight children globally, an increase of 8 million since 2000 [1]. The emergence of overweight and obesity is due to increasing access to processed foods and lower levels of physical activity [2]. Overweight or obesity in childhood increases the risk of morbidity and mortality in adulthood [2].

In Morocco, few studies on SS and obesity among school children have been performed, and no study on the prevalence of SS and obesity in Eastern Morocco has been undertaken.

Therefore, we aimed to estimate the prevalence of childhood SS, underweight, overweight, and obesity among primary school students of Oujda in Eastern Morocco. Knowledge of the prevalence of childhood SS, underweight, overweight, and obesity is important to inform the development of public health programs.

## Materials and methods

We conducted a cross-sectional observational study among primary schools in Oujda-Angad Province from November 2017 to April 2018, with a stratified random cluster sampling of public, private, urban, and rural primary schools based on the database provided by the statistical office of the Eastern Region Academy. The sampling unit was the school, and all students from the randomly selected schools were selected to participate in the survey.

The sample size was calculated using the following formula: N = k × x (1-) × (Za/p)2, where N is the sample size, k (cluster effect) = 2, Za (the expected proportion of childhood overweight and obesity) = 1.96, and p (allowable error of known prevalence) = 2%.

We obtained a representative sample that comprised 1960 students (1293 students from public sector schools and 667 students from private sector schools). In addition, students from the public sector schools were divided into two public subsectors: urban students (n = 873) and rural students (n = 420).

The study was approved by the Ethics Board of the Biomedical Research of Oujda. Moreover, formal authorizations were granted by the Ministry of Education and the schools' principals and teachers. The parents were asked to provide written informed consent. The students were informed about the objectives of the study, and most importantly, the students’ assent to participate was requested on the day the measurements were taken.

The children’s weight was measured to the nearest 0.1 kg using a standardized scale, and their height was measured to the nearest 0.1 cm using a portable stadiometer equipped with a movable headrest horizontal to the nearest millimeter.

We examined the children’s personal and family history using the child health booklet. Then, each student’s height, weight, and head circumference were measured. Body mass index (BMI) was calculated by dividing the body weight in kilograms by height in meters squared (kg/m^2^) and categorized according to the international standard cutoffs: underweight (<15th percentile), overweight (85th-95th percentile), and obese (>95th percentile).

In our study, we used two different references to classify the children’s status: 1) the age and sex reference standards established by the WHO in 2007, applicable to children aged five to 19 years and established from cross-sectional data from the 1977 National Center for Health Statistics (NCHS)/WHO references [3], and 2) the International Obesity Task Force (IOTF) growth charts [4] developed in 2000.

Data analysis was performed using SPSS version 21.0, with which descriptive statistics were calculated for all the variables (means, standard deviations, and percentages). Student’s t-test was used to compare the groups’ mean values, and the chi-square test was used to compare the percentages. For all the statistical tests, a p-value < 0.05 was considered significant.

## Results

Demographic data

A representative sample of 1582 students aged from five to 15 years old was selected from urban private, urban public, and rural public primary schools. The average age was 9.3 ± 1.9 years; 803 (50.8%) were males and 779 (49.2%) were females. The average age of the girls was 9.5 ± 1.9 years old, and for boys, it was 9.4 ± 2 years old. The largest proportion of the sample was students aged seven years old (n = 293; 18.9%).

**Figure 1:**
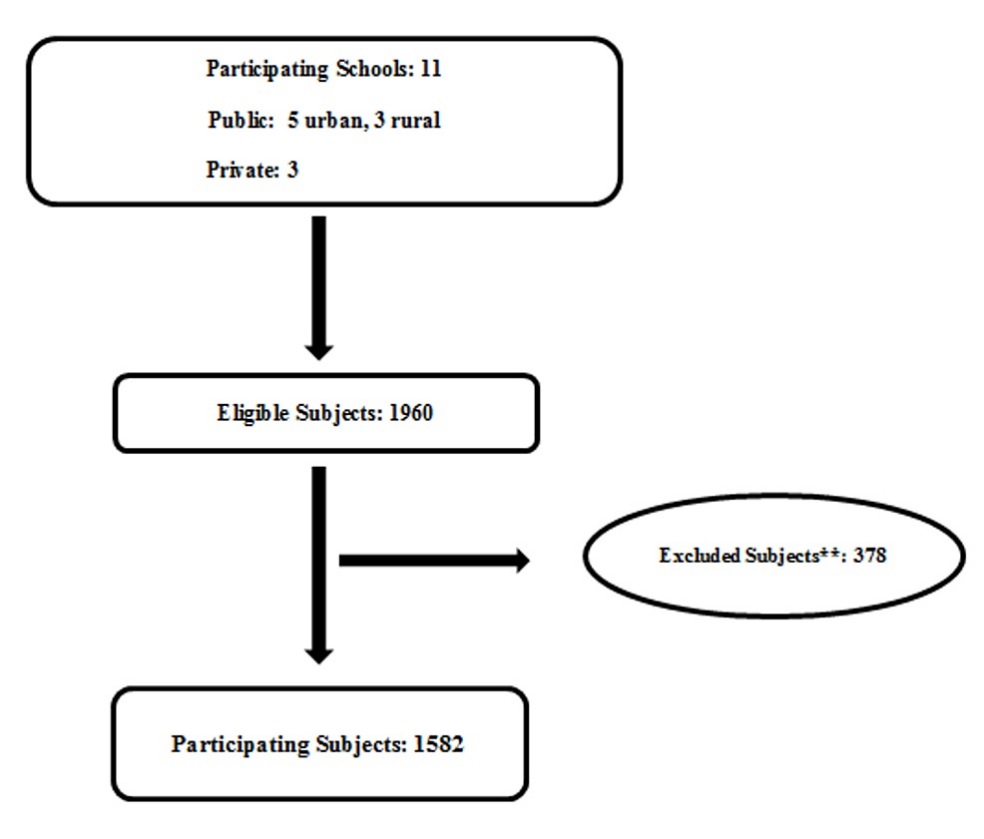
Flowchart demonstrating the recruitment of the schools and their students **Subjects excluded because of the lack of written parental consent

More than 90% of the study participants were from urban schools. The demographic distribution of the sample is illustrated in Figure 1.

Anthropometric data

Weight

The average weight of the students was 29.3 ± 9.6 kg (range: 12-130 kg). The average weight of the girls was 29.1 ± 9.7 kg (range: 14-85 kg), while the average weight of the boys was 29.6 ± 9.5 kg (range: 12-130 kg) (Table 1).

Height

The average height was 133.7 ± 12.2 cm (range: 104.5-175.5 cm). The girls’ average height was 133.3 ± 12.6 cm (range: 104-175 cm), while the boys’ average height was 134.4 ± 11.6 cm (range: 110-172 cm) (Table 1).

**Table 1:** Sociodemographic and anthropometric characteristics of the sample population BMI: body mass index, SD: standard deviation

Child characteristics	Male	Female	Total
Sex	803	779	1582
Height in cm (mean ± SD)	134.4 ± 11.6	133.3 ± 12.6	133.7 ± 12.1
Weight in kg (mean ± SD)	29.6 ± 9.5	29.1 ± 9.7	29.3 ± 9.6
BMI in kg/m² (mean ± SD)	16.1 ± 3.4	16.0 ± 3.1	16.0 ± 3.3

Body Mass Index

The mean (SD) BMI was 16.05 (3.3) kg/m^2^ (Table 1), with a minimum of 8.3 kg/m^2^ and a maximum of 76.9 kg/m^2^, showing a significant trend with the lowest value in the public schools (with the lowest socioeconomic status [SES]) and the highest value in the private schools (with the highest SES) (p < 0.001) (Table 2).

**Table 2:** Body mass index assessment of students according to school types

School types	Average	Minimum	Maximum	Standard deviation
Private	16.9	11.8	30.3	±2.9
Public	15.7	8.3	76.7	±3.4
Urban	16.0	8.3	76.9	±3.3
Rural	16.4	12.6	22.2	±2

Epidemiological data of growth disorders

The prevalence of SS, underweight, overweight, and obesity among children in primary schools are shown in Table 3.

**Table 3:** Prevalence of underweight, overweight, obesity, and short stature in children according to schools and sex using the World Health Organization standards

Demographic variables	Underweight		Obese		Short stature		Normal	Total
	n (%)	p	n (%)	p-trend	n (%)	p-trend	n (%)	
Private/public	2 (0.5)/50 (4.4)	<0.001	81 (18.6)/96 (8.5)	<0.001	2 (0.5)/33 (2.9)	0.003	356 (80.7)/962 (84.3)	441/1141
Urban/rural	51 (3.5)/1 (1.0)	0.146	170 (11.5)/7 (7.1)	0.17	33 (2.2)/2 (2.0)	0.619	998 (67.7)/89 (89.8)	1473/99
Male/female	19 (2.4)/33 (4.2)	0.04	80 (10.0)/97 (12.5)	0.116	20 (2.5)/15 (1.9)	0.444	684 (85.18)/634 (81.3)	803/779

Obesity was documented in 11.2% of the students. Overall, the prevalence of obesity was significantly different between private and public schools. Using the WHO standards [3], the prevalence of obesity was higher in the private sector schools with the highest SES students (18.6%) and lower in the public sector schools with the lowest SES students (8.5%) (p < 0.001), while the prevalence was 8.4% and 3.5%, respectively, using the International Obesity Task Force (IOTF) cutoffs [4]. Moreover, a higher prevalence of obesity was discovered among children enrolled in urban schools (11.5%), compared with rural schools (7.1%) using the WHO standards, without a statistically significant difference (p = 0.17).

An intergroup comparative analysis showed that obesity was much more pronounced in the pubescent students (25.2%) than in the non-pubertal students (8.7%) (p = 0.001). The BMI distribution of the different categories by sex showed that the proportions estimated by the two methods of overweight and obesity were higher among girls (12.5% versus 10%), but this difference was not significant (p = 0.116).

The same trend of a higher prevalence in private schools was found for overweight and obesity (p < 0.001). When we combined overweight and obese individuals in the analysis, we observed the highest frequency (20.6%) for the private schools (ranked as the highest SES), whereas the lowest prevalence (9.1%) was found in the public schools (lowest SES) (p = 0.001).

In contrast, the overall prevalence of underweight was 23%, which was significantly associated with the school strata (p = 0.001). Using the IOTF cutoffs, the private sector schools had a lower prevalence of 7.5% compared with the public sector, where the prevalence was 29%. These prevalence rates were 0.5% and 4.4%, respectively, using the WHO standards. However, there were no significant differences in the prevalence of underweight and stunting between the sexes.

Overall, SS was recorded among 2.2% of the total sample. The lowest frequency (0.5%) was observed in private schools and the highest frequency (2.9%) in public schools (p < 0.001). Sex was not associated with the prevalence of SS: 2.5% of the males had SS compared with 1.9% of the females. Moreover, 21% of the girls with SS (-2 SD) had an advanced Tanner stage of III, and 78% had Tanner stage I, while for boys with SS, 15% had a Tanner stage II, and 85% had Tanner stage I.

## Discussion

This study comparing the prevalence of growth disorders among children enrolled in private, public, urban, and rural primary schools in Oujda-Angad Province is the first of its kind in Morocco.

It is well documented that growth disorders are not a simple problem. Multiple determinants of their occurrence include direct determinants such as diet and health and indirect determinants such as families’ socioeconomic status, parents’ nutritional and educational status, and the availability of clean potable water and sanitary facilities, as well as the interactions of these determinants.

A detailed evaluation should be conducted to identify the cause of a child’s suspected impaired growth. Such an evaluation may include a combination of personal, family, and social history, physical examination, general and perhaps specialized laboratory evaluations, radiologic examinations, genetic testing, and consultation with an endocrinologist.

Unfortunately, the data collected for our study were limited, and there are no time trends or historical data on growth disorders for reference or comparison purposes. No data were collected on dietary behavior, dietary intake, nutrition, physical activity, psychosocial factors, transportation to school, television viewing, or family history of growth disorders, and birth weight was mostly not available.

Overall, we found remarkably different prevalence rates of growth disorders across school types, showing the impact of SES. Children in public schools are characterized more by a significant failure to thrive than their counterparts in private schools. Conversely, the risk of overweight and obesity characterizes children from private schools, where the socioeconomic level of parents rises from middle to high. This is borne out by the annual tuition fees per student of at least 8000 MAD, while public school education is free.

Stature

Our findings revealed that, among children in primary schools of Oujda, 2.2% had SS, with the highest prevalence in the public sector than in the private sector (2.9% versus 0.5%). An ongoing study conducted in England concluded that social differences were significantly associated with children’s height [5]. A total of 21% of the girls with SS had an advanced pubertal stage; however, their short stature was diagnosed late, and they will have a short adult height.

Conversely, the sex difference was not fundamentally connected with the prevalence of SS: 1.9% of the females had SS compared with 2.5% of the males. Prevalence rates based on sex are widely discussed in the literature, including a study conducted in nine European countries in which the SS prevalence rates were 2.8% and 1.4% among 11-year-old females and males, respectively [6].

Ponderal growth

Data concerning underweight are variable in the literature, with prevalence ranging from 6% in France to 24.5% in Algeria [7,8]. In our study, underweight occurred in 23% of the children, and the private school students had a lower prevalence of 7.5% compared with the public sector school students, where the prevalence was 29%.

The higher prevalence of SS and underweight in students at public schools can be explained by the parents’ lower SES, lower average income, and lower educational level. It could be argued that an individual’s SES, food insecurity, lower access to healthy foods, and inadequate education have major impacts on nutritional status [9].

Several related factors can also affect children’s growth rate, such as heredity, hormonal secretion, certain diseases, and inadequate caregiving. Repeated infections particularly affect children’s growth in low- and middle-income countries, where the incidence of these infections can be very high.

Another possible factor contributing to the patterns of SS and underweight distribution could be the rate of consanguineous marriages in Arab countries. Consanguinity is related to an increased likelihood of the spread of passive characteristics and diseases, some of which may antagonistically influence children’s height and weight [10].

Childhood obesity has been steadily increasing in recent decades in both industrialized and low- and middle-income countries, and several epidemiological studies have highlighted the role of obesity as an independent risk factor for cardiovascular disease [11].

The prevalence of overweight in our study was 11.2%, which is consistent with the findings from neighboring countries. Another study of overweight in children from 21 European countries utilizing the IOTF cutoff for overweight demonstrated that higher prevalence rates were found in Mediterranean countries such as Spain, Italy, and Greece among children between seven and 11 years old [12]. Unfortunately, this was because these countries as well as those of North Africa no longer follow the Mediterranean diet, having changed eating habits toward fast food and junk food that combine reduced prices, fat, and sugar.

Compared to industrialized countries, the prevalence of obesity in our study is lower. In high-income countries, obesity is at an alarming rate because of the high standard of living. This prevalence was 16.9% in the United States in 2010 [13]. The proportions for overweight and obesity are even higher in Kuwait in the Middle East, reaching 20.2% for overweight and 16.8% for obesity in boys aged six to 10 [14].

In our study, the prevalence of obesity was higher than that found in Nigeria by Senbanjo et al. (5.2%) in preschool children [15]. This difference could be explained by the choice of age group, which is wider in our study (five to 15 years).

Our study showed that the prevalence rate of overweight and obesity was higher in private schools than in public schools in the same province. The findings of the current study on the positive association between SES and BMI are consistent with previous studies from India and Indonesia [16,17]. This may be attributed to the fact that a high caloric diet and fast food are easily accessible to high SES groups compared with poor families in low- and middle-income countries.

In our study, a higher prevalence of obesity was also found in children enrolled in urban schools (11.5%) compared with rural schools (7%). Here, we can distinguish two factors related to childhood obesity: lifestyles (urban or rural) and parents’ SES. The difference in the prevalence between urban and rural environments may be caused by the fact that there are no opportunities to run and play outdoors in some urban areas because of parental perceptions of danger. Most school children spend much of their time watching television and/or using computers for schoolwork and other recreational activities. By contrast, rural children are solicited for agrarian work outside the classroom, which expends more energy compared with their urban peers. Rural schools are often remote, and physical effort is necessary because of the lack of school transport.

We found that girls were more overweight among all the children in primary schools than boys. This difference in prevalence both in urban and rural areas could be explained by the fact that, in general, boys have a greater disposition to engage in physical activity and so are much less sedentary than girls.

Other researchers have recognized parental overweight and obesity as the most significant determinant of childhood overweight [18,19]. Although genetic factors play a role in BMI and total body fat, evidence suggests that the shared environment is a much more important determinant of the high correlation between parental and child BMI [20].

The comparison of our results with the literature showed several differences in methodology, which prevented direct comparison. The differences observed between countries can be explained by the influence of the study area, the heterogeneity of the ages included, and above all the variety of norms adopted by the authors in their definition of low birth weight, overweight, and obesity. The use of either reference (IOTF or WHO) may lead to quite different estimates of prevalence, which requires careful interpretation of the results.

## Conclusions

Our study provides an estimate of the prevalence of obesity, overweight, underweight, and SS in Oujda-Angad Province, Morocco. Our results reflect the importance of the problem, the need to monitor the nutritional status at both the individual and the community level, and the need to put in place preventive, diagnostic, and early management strategies.

There is a critical requirement for the school health program to periodically monitor children’s dietary habits and physical development. Pertinent advice on nutrition and physical exercise ought to be given not exclusively to school children but additionally to their educators and parents or guardians.

## References

[1] (2020). Levels and trends in child malnutrition: UNICEF/WHO/The World Bank Group joint child malnutrition estimates: key findings of the 2020 edition. https://www.who.int/publications/i/item/jme-2020-edition.

[2] Reilly JJ, Kelly J (2011). Long-term impact of overweight and obesity in childhood and adolescence on morbidity and premature mortality in adulthood: systematic review. Int J Obes (Lond).

[3] de Onis M, Onyango AW, Borghi E, Siyam A, Nishida C, Siekmann J (2007). Development of a WHO growth reference for school-aged children and adolescents. Bull World Health Organ.

[4] Cole TJ, Bellizzi MC, Flegal KM, Dietz WH (2000). Establishing a standard definition for child overweight and obesity worldwide: international survey. BMJ.

[5] Hancock C, Bettiol S, Smith L (2016). Socioeconomic variation in height: analysis of National Child Measurement Programme data for England. Arch Dis Child.

[6] Yngve A, De Bourdeaudhuij I, Wolf A (2008). Differences in prevalence of overweight and stunting in 11-year olds across Europe: The Pro Children Study. Eur J Public Health.

[7] Rolland-Cachera MF, Castetbon K, Arnault N (2002). Body mass index in 7-9-y-old French children: frequency of obesity, overweight and thinness. Int J Obes Relat Metab Disord.

[8] Oulamara H, Agli AN, Frelut ML (2009). Changes in the prevalence of overweight, obesity and thinness in Algerian children between 2001 and 2006. Int J Pediatr Obes.

[9] Sultan M, Afzal M, Qureshi SM (2008). Etiology of short stature in children. J Coll Physicians Surg Pak.

[10] Galal OM (2002). The nutrition transition in Egypt: obesity, undernutrition and the food consumption context. Public Health Nutr.

[11] Hubert HB, Feinleib M, McNamara PM, Castelli WP (1983). Obesity as an independent risk factor for cardiovascular disease: a 26-year follow-up of participants in the Framingham Heart Study. Circulation.

[12] Lobstein T, Frelut ML (2003). Prevalence of overweight among children in Europe. Obes Rev.

[13] Ogden CL, Carroll MD, Kit BK, Flegal KM (2012). Prevalence of obesity and trends in body mass index among US children and adolescents, 1999-2010. JAMA.

[14] Al-Isa AN, Campbell J, Desapriya E (2010). Factors associated with overweight and obesity among Kuwaiti elementary male school children aged 6-10 years. Int J Pediatr.

[15] Senbanjo IO, Adejuyigbe EA (2007). Prevalence of overweight and obesity in Nigerian preschool children. Nutr Health.

[16] Tharkar S, Viswanathan V, Thomas O (2009). Impact of socioeconomic status on prevalence of overweight and obesity among children and adolescents in urban India. Open Obes J.

[17] Julia M, van Weissenbruch MM, de Waal HA, Surjono A (2004). Influence of socioeconomic status on the prevalence of stunted growth and obesity in prepubertal Indonesian children. Food Nutr Bull.

[18] Strauss RS, Knight J (1999). Influence of the home environment on the development of obesity in children. Pediatrics.

[19] Danielzik S, Czerwinski-Mast M, Langnäse K, Dilba B, Müller MJ (2004). Parental overweight, socioeconomic status and high birth weight are the major determinants of overweight and obesity in 5-7 y-old children: baseline data of the Kiel Obesity Prevention Study (KOPS). Int J Obes Relat Metab Disord.

[20] Trasande L, Cronk C, Durkin M (2009). Environment and obesity in the National Children's Study. Environ Health Perspect.

